# To Pursue or Not to Pursue an Academic Doctoral Degree: Perceived Motivators and Barriers of Women Working in Allied Health Educational Programs

**DOI:** 10.1007/s40670-026-02709-4

**Published:** 2026-03-31

**Authors:** Karen Honeycutt, Betsy Becker, Victoria Kennel, Sarah McBrien, Makayla Schissel, Tammy Webster

**Affiliations:** 1 CAHP Clinical, Diagnostic, & Therapeutic Sciences, University of Nebraska Medical Center, Omaha, NE USA; 2 CAHP Health & Rehabilitation Sciences, University of Nebraska Medical Center, Omaha, NE USA; 3 CAHP Education, Research & Practice, University of Nebraska Medical Center, Omaha, NE USA; 4 COPH Biostatistics, University of Nebraska Medical Center, Omaha, NE USA; 5 Medical Sciences, University of Arkansas, Fayetteville, AK USA

**Keywords:** Allied health, Women faculty, Higher education, Career development, Academic doctorate

## Abstract

**Background:**

Women faculty in allied health education frequently transition from clinical roles without formal training in teaching or research methodologies. This study examined motivators and barriers of women faculty in an allied health educational setting when determining to pursue an academic doctoral degree. Data was collected from faculty working in 48 allied health programs in Nebraska representing 14 professions.

**Methods:**

Women faculty were invited to complete a confidential online survey. Using a four-point Likert scale, participants responded to perceived motivators (nine) and barriers (nine) related to their decision to pursue or not pursue a doctoral degree.

**Results:**

Responses from 142 (49.6%) surveys were examined. Participants were grouped as not having applied and those who were pursuing or holding a PhD/EdD degree. Comparing the two cohorts, statistically significant differences (p ≤ 0.05) were found for the motivators: positively impact professional relationships, expand professional network, and expand research knowledge; and the barriers: time to complete a doctoral degree program, the day-to-day commitments, personal financial cost, and impact on personal relationships. Multivariable logistic regression analysis indicated that age, academic rank, perceived barriers, and perceived motivators were all significantly associated with pursuing or attaining an academic doctoral degree.

**Conclusions:**

Identifying perceived motivators can inform strategies in supporting a women faculty’s career with professional network development and research opportunities. Addressing barriers through work-life integration support and financial assistance may improve doctoral program accessibility.

## Introduction

Women faculty in allied health education often transition from clinical practice to academia without formal training in teaching, learning, or research methodologies—skills essential for success in academic roles. Academic requirements for allied health faculty vary across programs. Several allied health educational programs, such as cytology, medical imaging, and medical laboratory science, adhere to programmatic accreditation standards which require only the program director to have an earned master’s degree or higher [1–6]. Per clinical perfusion accreditation standards, the program director's minimal educational requirement is a master’s degree [7].This differs from non-health-related academic faculty positions that highly encourage an academic doctorate or an entry-level professional doctoral degree. Educational accrediting bodies for allied health professions have developed requirements for faculty in educational programs to hold a professional or academic doctoral degree. For example, physical therapy accreditation requires all core faculty to have some type of doctoral preparation and the program director to have an academic doctoral degree [8]. In the physician assistant profession, where the terminal degree to practice is at the master’s level, the minimum requirement for a program director is a master’s degree, but the number of educational program directors holding a doctoral degree increased by nearly 2% from 2017 to 2019 [9, 10]. Academic doctoral degree requirements may have an impact on how women faculty in health professions educational programs can be qualified to meet career goals as faculty.

Currently in higher education, women make up the majority of nontenure-track lecturers and instructors. [11] Despite greater numbers of women in medicine, more men than women secure senior positions [12]. Per Bond et al. [13], between 1989 and 2009, females in pharmacy academia more than doubled. Yet, women are still disadvantaged in pharmacy academia compared to White men related to administrative ranks [13]. This invites the question of the preparedness of women in medicine, and for this study in allied health educational programs, to assume leadership opportunities. What educational and professional attributes will enable women to achieve parity in academic leadership roles? If more higher education administrative and leadership positions require an academic doctorate (i.e., PhD, EdD), women without these credentials will not be eligible for advancement into these opportunities [14, 15]. A major focus of an academic doctoral degree is developing research skills needed for faculty scholarship expectations [16, 17]. For those moving from clinical practice to an academic role, earning an academic doctorate may provide more insight into the expectations of higher education faculty. Earning a doctoral degree enables women to serve as influential role models for colleagues who have not yet attained this level of education.

Deciding to pursue doctoral studies can be difficult and intimidating. For working women in academia, work-life integration can be tough enough without adding the challenges that come with completing a doctoral-level program of study [14]. The purpose of this research is to investigate the motivations and barriers to earning an academic doctoral degree as perceived by women working in allied health educational programs, with a specific focus on comparing those with and without an academic doctoral degree. Identifying overarching hypotheses related to motivations and barriers that influence a woman’s decision to pursue an academic doctorate will provide information to better develop and leverage institutional support and mentorship resources. The research was approved by the University of Nebraska Medical Center IRB (#0598–21-EX).

### Theoretical Framework

The study’s conceptual framework is based on the social-ecological model that states the actions of individuals and groups are mediated by ever-changing social contexts influenced by the interacting domains of individual, micro- and macro-environmental systems [18, 19]. The social-ecological model is a framework previously used to understand individual decision-making and responses in areas such as personal well-being, education, health, career, and family [20–24].

The social-ecological model defines how the complex interactions between factors related to an individual, their relationships, their communities, and societal contexts impact one’s lived experience and decision-making processes. The model expands from individual, micro, and macro-levels of influence [18, 19]. The first level considers an individual’s characteristics, psychosocial tendencies, knowledge, and skills. An individual’s intrinsic and extrinsic motivations related to life-long learning inclinations or earning power expectations might impact their decision to continue their education. The model’s next level considers a micro-environment around relationships within the home, school, and workplace. How an individual’s job is defined and their roles and responsibilities within these various complex relationships may impact the decision to take on additional commitments and duties within a doctoral program. The model’s third level considers the macro-environmental influences related to different communities, such as health care, higher education, and academic institution groups. The perceived value of earning an academic doctorate or the influences related to the promotion and tenure progression in these various communities may impact a woman’s decision-making process. The final macro-level is societal and cultural norms and beliefs. The status and recognition within various culture systems may impact one’s decision to earn a doctoral degree.

## Methods

A convenience, non-probability sampling [25] was used to identify potential participants as women faculty working within a Nebraska accredited allied health educational program. Allied health professions were defined as non-nurse, non-pharmacy, and non-physician health care providers [26]. Programs and their institution websites were identified through public, online accreditation agency databases. From this search, 48 educational programs were identified representing 14 allied health professions. Educational institutions represented included health care organizations (i.e., hospital-based programs), community colleges, public and private four-year colleges or universities, and public and private academic medical centers. From publicly available institution websites, 286 individuals were identified as female based on their names, website images, or the research team’s professional networking relationships in the region, and if there was any ambiguity, the individual was invited to complete the survey. To validate gender identity, a survey question asked participants if they identify as: agender, cisgender, genderqueer, man, nonbinary, transgender, woman, other or prefer not to answer.

The confidential, online survey included two question groups. Group one included categorized constructs of nine motivators and nine barriers for making the decision to pursue an advanced degree (i.e., an EdD or PhD) through an academic doctoral program. The motivators and barriers used in the survey were identified from prior studies that explored the experiences of women throughout the journey of earning a doctoral degree [27–31] and anecdotes from the authors and those considering, completing, or that have already earned this educational milestone. See Table 1 for motivators and barriers included in the survey. The second group of questions captured participant socio-demographic information, which included whether the participant completed, was pursuing, had applied or had not applied to an academic doctoral program. Before disseminating the survey to the eligible participant pool, it was first piloted by eight women who met inclusion criteria. Based on pilot participant feedback, wording of some survey questions was revised to improve clarity for the final version.

**Table 1:** Survey motivators and barriers

Motivators* I decided to pursue (or am considering pursuing) my EdD or PhD because I wanted (or want) to:	Barriers* I was (or am) apprehensive to pursue my EdD or PhD because I was (or am) concerned about:
• Improve personal growth • Improve my professional opportunities • Increase my career advancement opportunities • Increase my salary • Increase my professional status and influence • Positively impact my professional relationships (e.g., colleagues, collaborators, supervisors) • Expand my professional network • Expand my research and scholarly activity knowledge • Be a role model for other women in academia	• Finding information from academic institutions about potential doctoral programs • Making a formal commitment to a doctoral program • How long it would take me to finish a doctoral program • The day-to-day (day-to-night) time commitments • The personal financial cost • My academic ability to complete a doctoral program • Support from my employer related to completing a doctoral program • Impact on my professional relationships (e.g., colleagues, collaborators, supervisors) • Impact on my personal relationships (e.g., parental, significant others, friends)

^*^Likert scale used: Strongly Disagree = 1 Disagree = 2 Agree = 3 Strongly Agree = 4

Qualtrics (Provo, UT) survey software was used to develop and administer the survey. Two days prior to releasing the confidential, online survey, a pre-notice invitation to complete the survey was emailed to the 286 identified faculty using their professional email addresses. Subsequently, over a two-week period, after which the survey link was provided, invitees that had not submitted their survey responses received up to three reminders.

Descriptive statistics (means, standard deviations, medians, counts, and percentages) were used to summarize the demographics and survey responses between groups and for the total responses. Survey participants were asked to select their level of agreement using a four-point Likert Scale (i.e., strongly agree, agree, disagree, strongly disagree) across several motivators and barriers when pursuing or deciding to pursue a doctoral degree. For analysis purposes, strongly agree and agree responses were grouped together and strongly disagree and disagree responses were grouped together. Chi-Square or Fisher’s Exact test were used as appropriate to compare the various motivators and barriers between those Pursuing/Attained a doctoral degree and those who have Not Applied to a doctoral degree program.

To reduce dimensionality and account for conceptual overlap among motivator and barrier items, an exploratory factor analysis (EFA) was performed on the significant motivators and barriers in univariate analysis using principal factor extraction (method = principal) with promax (oblique) rotation to allow correlation between factors. The number of factors retained was two, based on scree plot inspection and eigenvalues greater than one. Factor scores were calculated for each participant and saved for use in subsequent regression analyses. Factor 1 predominantly represented barriers (program duration, time commitment, financial cost, personal relationship impact), while Factor 2 predominantly represented motivators (professional relationships, networking, research knowledge). Multivariable logistic regression was used to assess the association between the latent motivator and barrier factors and PhD pursuit/attainment. Factor 1 (barriers) and Factor 2 (motivators) were included as predictors. Age and current academic rank were measured at the time of survey administration and were included as covariates to account for career stage. Because academic rank may be influenced by doctoral attainment, analyses including rank were considered exploratory. The outcome of interest was the odds of being in the “Pursuing/Attained” group. Adjusted odds ratios (aORs) with 95% confidence intervals (CIs) were estimated. All analyses were conducted in SAS version 9.4. *P* < 0.05 was considered statistically significant.

## Results

A total of 150 individuals participated in the survey (52% response rate). Seven participants were removed due to incomplete survey responses. One additional participant was removed as they had applied to a doctoral degree program, while our particular interest revolved around comparing individuals who were pursuing or attained a doctoral degree with individuals who had not attained a doctoral degree. Of the 142 total analyzable participant survey responses, 39.4% (n = 56) are pursuing or have attained a doctoral degree at the time of survey participation. In total, 14 allied health professions were represented. [26] Participants were primarily from the allied health fields of occupational therapy (16.7%), physical therapy (16.7%), the medical imaging and therapeutic sciences (10.1%), and medical laboratory science (8.7%). Most participants (58.9%) in the Pursuing/Attained group are in faculty roles, while the majority (39.5%) of those in the Not Applied group have multiple roles such as clinical coordinator and faculty, clinical faculty and faculty, and faculty and program director. There is a similar representation of first-generation college graduates in both groups (Not Applied: 41.9%, Pursuing/Attained: 37.5%). For survey participant demographics, see Table 2.

**Table 2:** Survey socio-demographics

	Group		
	Not applied (*N* = 86)	Pursuing/Attained (*N* = 56)	Total (*N* = 142)
My present role(s) at my current academic employer is(are): *n* (%)			
Faculty	26 (30.2%)	33 (58.9%)	59 (41.5%)
Program director	9 (10.5%)	5 (8.9%)	14 (9.9%)
Clinical coordinator and faculty	7 (8.1%)	3 (5.4%)	10 (7.0%)
Clinical faculty and faculty	7 (8.1%)	1 (1.8%)	8 (5.6%)
Clinical faculty	7 (8.1%)	1 (1.8%)	8 (5.6%)
Administrator	3 (3.5%)	3 (5.4%)	6 (4.2%)
Administrator and faculty	2 (2.3%)	4 (7.1%)	6 (4.2%)
Program director and faculty	5 (5.8%)	1 (1.8%)	6 (4.2%)
Other	7 (8.1%)	3 (5.4%)	10 (7.0%)
Other: Multiple roles	13 (15.1%)	2 (3.6%)	15 (10.6%)
My current academic appointment is best described as: *n* (%)			
Full-time (12 months)	76 (88.4%)	41 (73.2%)	117 (82.4%)
Full-time (9 months to 11 months)	5 (5.8%)	14 (25.0%)	19 (13.4%)
Part-time	5 (5.8%)	1 (1.8%)	6 (4.2%)
My current academic rank: *n* (%)			
Instructor/lecturer	23 (29.9%)	5 (8.9%)	28 (21.1%)
Assistant professor	39 (50.6%)	18 (32.1%)	57 (42.9%)
Associate professor	6 (7.8%)	19 (33.9%)	25 (18.8%)
Professor	3 (3.9%)	13 (23.2%)	16 (12.0%)
Other	6 (7.8%)	1 (1.8%)	7 (5.3%)
Missing	9 (10.5%)	0 (0.0%)	9 (6.3%)
I am a first-generation college graduate. *n* (%)			
Yes	36 (41.9%)	21 (37.5%)	57 (40.1%)
No	50 (58.1%)	35 (62.5%)	85 (59.9%)

Several motivators and barriers regarding the pursuit of an academic doctoral degree were compared between groups. Agreement in the prompt “Positively impact my professional relationships” significantly differed between groups (*p* = 0.006), where 65.9% of those in the Not Applied group agreed that this would be a motivator, compared to 87.0% of those in the Pursing/Attained group. Only 56.5% of individuals in the Not Applied group agreed that the expansion of their professional network would be a motivator to pursuing an academic doctoral degree, compared to 81.5% of those in the Pursuing/Attained group (*p* = 0.002). There was also a significant difference in agreement between whether the expansion of research or scholarly activity would be a motivator for pursuing an academic doctoral degree (*p* = 0.002). The overwhelming majority (96.4%) of individuals in the Pursuing/Attained group agreed with that statement compared to 76.5% of the Not Applied group. Other motivator noteworthy findings where both groups agreed (average for both groups > 89%) that pursuing a doctoral degree would improve personal growth (92.3%), improve professional opportunities (93.5%), and increase career advancement opportunities (89.9%). See Table 3 for all motivator data.

**Table 3:** Survey results for motivators

	Group			
	Not applied (*N* = 86)	Pursuing/Attained (*N* = 56)	Total (*N* = 142)	*P*-value
Improve my personal growth, *n* (%)				0.09^1^
Disagree	9 (10.6%)	1 (1.8%)	10 (7.1%)	
Agree	76 (89.4%)	54 (98.2%)	130 (92.9%)	
Improve my professional opportunities, *n* (%)				0.15^1^
Disagree	8 (9.4%)	1 (1.9%)	9 (6.5%)	
Agree	77 (90.6%)	53 (98.1%)	130 (93.5%)	
Increase my career advancement opportunities, *n* (%)				0.08^1^
Disagree	12 (14.1%)	2 (3.7%)	14 (10.1%)	
Agree	73 (85.9%)	52 (96.3%)	125 (89.9%)	
Increase my salary, *n* (%)				0.86^2^
Disagree	20 (23.5%)	12 (22.2%)	32 (23.0%)	
Agree	65 (76.5%)	42 (77.8%)	107 (77.0%)	
Increase my professional status and influence, *n* (%)				0.59^2^
Disagree	24 (28.2%)	13 (24.1%)	37 (26.6%)	
Agree	61 (71.8%)	41 (75.9%)	102 (73.4%)	
Positively impact my professional relationships, *n* (%)				***0.006***^**2**^
Disagree	29 (34.1%)	7 (13.0%)	36 (25.9%)	
Agree	56 (65.9%)	47 (87.0%)	103 (74.1%)	
Expand my professional network, *n* (%)				***0.002***^**2**^
Disagree	37 (43.5%)	10 (18.5%)	47 (33.8%)	
Agree	48 (56.5%)	44 (81.5%)	92 (66.2%)	
Expand my research and scholarly activity knowledge, *n* (%)				***0.002***^**1**^
Disagree	20 (23.5%)	2 (3.6%)	22 (15.7%)	
Agree	65 (76.5%)	53 (96.4%)	118 (84.3%)	
Be a role model for other women in academia, *n* (%)				0.19^2^
Disagree	24 (28.2%)	10 (18.5%)	34 (24.5%)	
Agree	61 (71.8%)	44 (81.5%)	105 (75.5%)	

^1^Fisher exact *p*-value

^2^Chi-square *p*-value

Approximately 77.6% of the Not Applied group agreed that the time it would take to complete an academic doctoral degree program would be a barrier to their pursuit, compared to 71.1% of those in the Pursuing/Attained group (*p* = 0.04). A higher percentage of individuals in the Not Applied group agreed that the day-to-day (day-to-night) time commitments would be a barrier to their pursuit of a doctoral degree (88.2% vs. 70.9%, *p* = 0.01). There is also a significant difference between groups in the level of agreement when considering the personal financial cost as a barrier to pursuing a doctoral degree (Not Applied: 77.6%, Pursuing/Attained: 56.4%, *p* = 0.008). Finally, 75% of respondents in the Not Applied group agreed that the impact on their personal relationships would be a barrier to pursuing a doctoral degree compared to 58.2% of respondents in the Pursuing/Attained group (*p* = 0.03). Another noteworthy barrier finding where both groups agreed (average for both groups > 79%) that academic ability was not a barrier in completing a doctoral program. See Table 4 for all barrier data.

**Table 4:** Survey results for barriers

	Group			
	Not applied (N = 86)	Pursuing/Attained (*N* = 56)	Total (*N* = 142)	*P*-value
Finding (adequate) information from academic institutions about potential doctoral programs, n (%)				0.26^1^
Disagree	68 (80.0%)	48 (87.3%)	116 (82.9%)	
Agree	17 (20.0%)	7 (12.7%)	24 (17.1%)	
Making a formal commitment to a doctoral program, *n* (%)				0.21^1^
Disagree	45 (52.9%)	35 (63.6%)	80 (57.1%)	
Agree	40 (47.1%)	20 (36.4%)	60 (42.9%)	
How long it would take me to finish a doctoral program, *n* (%)				***0.04***^**1**^
Disagree	19 (22.4%)	21 (38.9%)	40 (28.8%)	
Agree	66 (77.6%)	33 (61.1%)	99 (71.2%)	
The day-to-day (day-to-night) time commitments, *n* (%)				***0.01***^**1**^
Disagree	10 (11.8%)	16 (29.1%)	26 (18.6%)	
Agree	75 (88.2%)	39 (70.9%)	114 (81.4%)	
The personal financial cost, *n* (%)				***0.008***^***1***^
Disagree	19 (22.4%)	24 (43.6%)	43 (30.7%)	
Agree	66 (77.6%)	31 (56.4%)	97 (69.3%)	
My academic ability to complete a doctoral program, *n* (%)				0.27^1^
Disagree	70 (82.4%)	41 (74.5%)	111 (79.3%)	
Agree	15 (17.6%)	14 (25.5%)	29 (20.7%)	
Support from my employer related to completing a doctoral program, *n* (%)				0.09^1^
Disagree	55 (64.7%)	43 (78.2%)	98 (70.0%)	
Agree	30 (35.3%)	12 (21.8%)	42 (30.0%)	
Impact on my professional relationships, n (%)				0.75^2^
Disagree	79 (92.9%)	50 (90.9%)	129 (92.1%)	
Agree	6 (7.1%)	5 (9.1%)	11 (7.9%)	
Impact on my personal relationships, *n* (%)				***0.03***^**1**^
Disagree	21 (24.7%)	23 (41.8%)	44 (31.4%)	
Agree	64 (75.3%)	32 (58.2%)	96 (68.6%)	
Impact on my personal health wellbeing, *n* (%)				0.06^1^
Disagree	20 (23.5%)	21 (38.2%)	41 (29.3%)	
Agree	65 (76.5%)	34 (61.8%)	99 (70.7%)	

^1^Chi-square *p*-value

^2^Fisher exact *p*-value

Results from the multivariable logistic regression analysis indicated that age, academic rank, perceived barriers, and perceived motivators were all significantly associated with pursuing or attaining an academic doctoral degree.

Increasing age was associated with lower odds of pursuing or attaining a doctoral degree, with each additional year corresponding to a 5.7% decrease in the adjusted odds (aOR = 0.94; 95% CI: 0.89–0.999). Academic rank showed a strong relationship with doctoral pursuit. Compared with instructors/lecturers, associate professors had 26.2 times the odds (95% CI: 4.85–141.16) and professors had 15.4 times the odds (95% CI: 2.24–105.22) of pursuing or having attained a doctoral degree. Perceived barriers and motivators also played an important role. For each 1‑unit increase in the barriers factor score (reflecting stronger agreement with barriers), the odds of pursuing or attaining a doctoral degree decreased by 44% (aOR = 0.56; 95% CI: 0.33–0.95). Conversely, each 1‑unit increase in the motivators factor score was associated with a 2.6‑fold increase in the odds of pursuing or attaining a doctoral degree (95% CI: 1.43–4.60). See Table 5 for all multivariable logistic regression analysis.

**Table 5:**
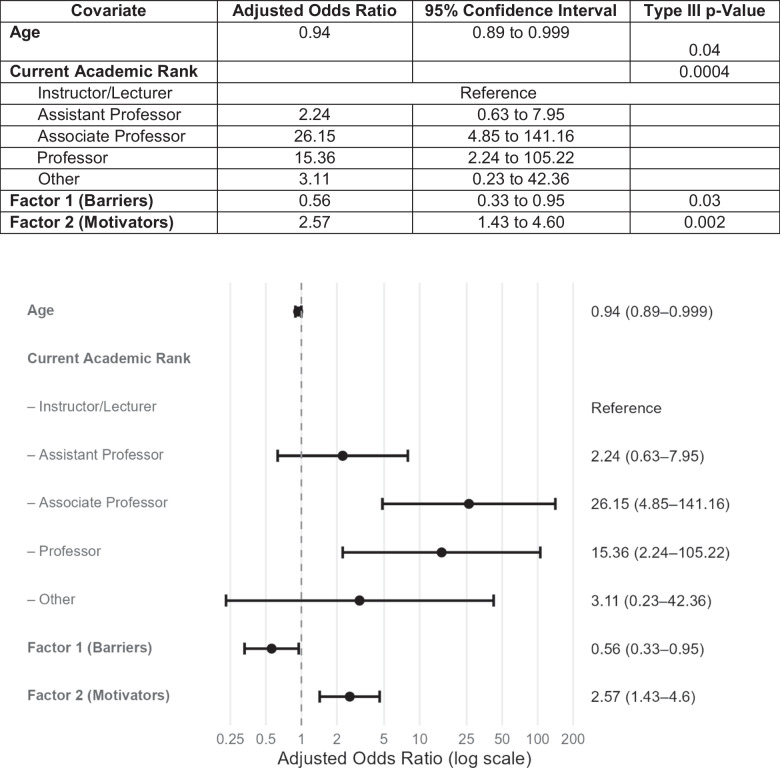
Multivariable logistic regression analysis data

## Discussion

**Fig. 1:**
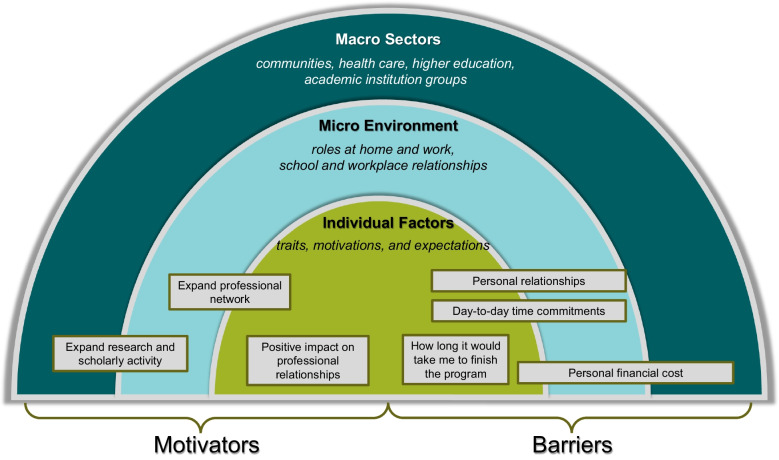
Statistically significant difference between the two groups embedded in the social-ecological model

**Fig. 2:**
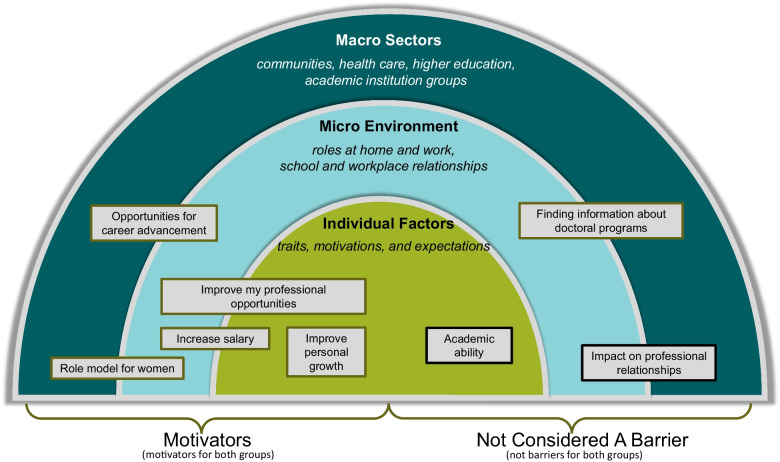
Agreement between the two groups about which factors are motivators and which are not barriers embedded in the social-ecological model

This study explored the perceived motivators and barriers of earning an academic doctoral degree by women allied health faculty. The comparison between women with and without an academic doctoral degree served as the primary study’s focus, aimed at generating hypotheses to guide educational institutions in better supporting allied health faculty. The social-ecological model postulates the actions of an individual are mediated by ever-changing social contexts influenced by the interacting domains of individual, micro- and macro-environmental systems. [18, 19] Employers’ awareness of the individual, workplace, and social norm elements and complex interactions impacting a woman’s decision to earn a doctoral degree, can provide guidance in supporting faculty (Figs. 1 and 2).

### Socio-Demographics

Compared with instructors and lecturers, our results show individuals at the associate professor and professor ranks had substantially higher odds of having pursued or completed a doctoral degree. This finding reflects an academic progression model in which advanced degrees may be prerequisites for promotion and expanded professional opportunities. Age emerged as an important predictor, with older respondents demonstrating lower odds of doctoral pursuit. Although the magnitude of this association was modest, the 5.7% reduction in odds per additional year suggests that faculty may perceive diminishing returns associated with undertaking doctoral training later in their careers. This may suggest early career mentorship and support can be impactful.

### Motivators

Stronger perceived motivators were associated with more than double the odds of doctoral pursuit, emphasizing the importance of factors such as improving professional growth, networks and scholarship agendas.

#### Improving Professional Growth and Networks

The Pursuing/Attained group saw motivators as positively impacting professional relationships (*p* = 0.006) and expanding professional networks, while the Not Attained group results were statistically significantly lower (*p* = 0.002). Both groups agreed that a motivator for earning a doctoral degree is improving professional opportunities (93.5%) and increasing career advancement opportunities (89.9%). These motivators impact the micro-environment of work roles. As employers of allied health profession women faculty, what strategies can be used to support the message and reality that earning an academic doctorate degree can provide meaningful, foundational networking opportunities to continue professional development to leadership roles? Carr et al. (2016) noted that more women in medicine is not translating to more achieved women leadership positions. [12] To address these gender equity issues, some medical institutions are incorporating formal, strategic programing for women faculty to help advance their careers. As a part of these programs, mentorship and networking target several levels within the social-ecological model. Mentoring was described as a cornerstone for promoting women faculty as was encouraging strong networks. [12] Chen et al. [32] describes a formalized pathway to develop future health professions educators. Learners identified engagement with the educator community of practice as the most powerful aspect of the pathway. [32] While in an academic doctorate program, teaching networking skills and elevating its importance, and providing such opportunities needs to be addressed by the educational program and/or the employer. These skills and relationships, which also can contribute to a formalized leadership succession plan, should be strategically developed, not expected to come to fruition by happenchance.

#### Research and Scholarly Agenda

Another area of statistically significant disagreement between the two groups is the Pursuing/Attained group agreed that a motivator to earning an academic doctorate is expanding research and scholarly activity knowledge (*p* = 0.002). Most allied health faculty, especially those working at four-year institutions, are expected to add to the professional discourse. Having the foundational skills to engage in scholarly endeavors is, for some, an essential skill to attain to meet requirements for academic promotion and continuous appointment/tenure. [33] Expanding research and scholarly activity impacts both the macro- and micro sectors. Connecting to the importance of professional network development, at the workplace and academic community, Becker et al. [34] describes junior physical therapy faculty scholarship productivity is influenced by the type of professional networks that are developed [34].

### Barriers

Overall, higher perceived barriers significantly decreased the likelihood of pursuing or attaining a doctoral degree, suggesting that time requirements and financial commitment-related challenges may deter faculty from engaging in advanced academic training.

#### Time

Time to complete the program (77.6%) and the day-to-day amount of time required for assignment completion (88.2%) were identified as barriers by those participants who have not applied to a graduate program. This concern may be further supported by the fact that 75.3% (n = 64) of participants who have not applied to a program cited the impact of pursuing a doctoral degree on their personal relationships as a barrier. Previous work done by this author group and by Brown and Watson [35] supports this finding that the time commitment required to complete an advanced degree program is a primary factor for women who are considering doing so. [35] Institutions that *employ* women who are enrolled in an advanced degree program may consider formally accommodating their needs by allowing modified, flexible, or abbreviated work schedules, providing extended childcare services, considering formal release from clinical or teaching duties, and implementing sabbatical opportunities during certain stages of the educational program.

Institutions where these academic programs are *offered* may further facilitate degree completion by arranging for classes to be delivered via remote or distance technologies, arranging some or all coursework to be completed asynchronously, and designing course assignments so that students may coordinate work tasks with course assignments when possible. Allied health professions educators bring experience in clinical education, interprofessional education, simulation, financial and affiliation agreements with health systems, leadership, licensure and other areas. By creating an environment in which women allied health faculty can earn an advanced education or business degree, for example, the institution benefits from the health professions educator’s diverse experiences, creating a stimulating environment for all learners.

#### Financial Commitment

Participants in the Not Applied group (n = 66, 77.6%) identified personal financial cost as a barrier to pursuing a degree more than twice the times as those in the Pursuing/Attained group (n = 31, 56.4%). Further investigation into the characteristics of the participants themselves (ie, age, career stage, role) and those participants' employment setting and associated tuition remission/forgiveness opportunities may explain the differences in the two groups. Regardless, 97 of 140 participants reported personal financial cost as a barrier, which should signal to institutions that are not offering tuition support to their employees the importance of reducing the financial burden of participating in a doctoral degree program for employees.

#### Academic Ability

No statistical difference existed (*p* = 0.27) between the two groups’ perceived academic ability being a barrier to completing a doctoral program with 82.4% of those who have not applied and 74.5% of those who are pursuing or have attained a doctoral degree disagreeing that academic ability was a barrier to pursuing a doctoral degree. There was not a statistically significant difference between the two groups for support from one’s employer (*p* = 0.09), finding adequate information about doctoral programs (*p* = 0.27), impact on professional relationships (*p* = 0.75), and making a formal commitment (*p* = 0.21) as barriers.

These results together may suggest that latent traits, such as motivation, in complex concert with external factors, such as time, financial resources, and one’s household and family obligations, may be too challenging to overcome for some female allied health professions educators without significant intervention from their employing and educating institution.

## Limitations

Due to the study’s limitations, the results should be used to develop general hypothesis that needs further investigation and exploration. The study was not designed to survey individuals who identify as male, therefore no findings can be inferred as to how motivators and barriers are the same or different for men as compared to women. It was not our intention to compare men and women; rather it was our intention to compare women who have and who have not pursued an advanced degree. Hypotheses about the factors that motivate and/or prevent men from pursing advanced schooling should not be made from this study.

The non-randomized selection of faculty from accredited Nebraska allied health programs can result in biases not accounted for in the research study. Caution should be used when generalizing the findings to other health profession educators and from different states and regions in the United States. The way individuals were identified as female for potential participation in the survey could have biased results and missed possible participants that met inclusion criteria.

The list of barriers and motivators was determined based on our prior work, existing literature, and socio-ecological framework and were presented to participants as they completed the survey. It is, therefore, possible that there are other factors that act as motivators and barriers that were not included in the survey, such as specific life circumstances and challenges. A systematic review of qualitative responses to items on the survey asking for explanations for survey responses may shed light on the intersectionality of the motivators, the barriers, demographics of the participant, and their personal circumstances.

The survey did not include definitions of the terms used in the survey (e.g. academic ability, professional relationships, personal relationships). This may have led to differences in interpretation of the survey items. For instance, academic ability is an ill-defined construct generally, and we did not provide a definition with the survey. Some participants may have perceived this to be strictly the skills needed to complete course work, whereas others may have incorporated some of their own perceptions about test-taking or time management in developing their own definition of academic ability. In future studies, definitions of key construct language could include definitions for participant clarity.

The Pursing/Attained group’s perceptions and experiences of their doctoral program can change their motivator and barrier perceptions as compared to those that have not experienced a doctoral educational program. The time between doctoral and survey completion, with differences in professional experiences, can also change motivator and barrier perceptions. In future research, more connections can be made with participant subgroup similarities, such as academic rank, appointment type, first-generation status and motivators/barriers.

## Conclusion

Women faculty in allied health education identified motivators and barriers that shape their decisions to pursue an academic doctoral degree. Those who had pursued or attained a doctorate reported stronger motivators, particularly the desire to enhance professional relationships, expand networks, and strengthen research skills, while those who had not applied perceived significantly greater barriers related to time demands, financial cost, and the impact on personal relationships. Age, academic rank, and perceived motivators and barriers were meaningfully associated with doctoral pursuit, underscoring the influence of career stage and contextual factors on pathways to advanced study.

These findings carry important implications for health professions educational administration. Institutions play a critical role in reducing structural barriers by offering flexible work arrangements, financial support, and accessible educational pathways. Equally essential are intentional mentorship and networking opportunities that help women faculty envision the long-term value of doctoral preparation. By fostering environments that strengthen motivators and mitigate barriers, academic and employing institutions can better support women in pursuing advanced degrees, ultimately contributing to a more robust, research prepared, and leadership-ready allied health faculty workforce.
